# A Contemporary Geographical Perspective on Embodied Non‐Belonging: Migrant Health and Implications for Nursing Research

**DOI:** 10.1111/nin.70188

**Published:** 2026-11-15

**Authors:** Julien Richard, Gavin J. Andrews

**Affiliations:** ^1^ School of Nursing, Faculty of Health Sciences University of Ottawa Ottawa Ontario Canada; ^2^ Department of Health, Aging & Society McMaster University Hamilton Ontario Canada

**Keywords:** affect theory, belongingness, human geography, nursing, posthumanism, relational space

## Abstract

This article proposes an innovative way of understanding health inequities among migrant populations that moves beyond approaches based on social determinants of health or fixed categories, advancing an ontological and epistemological shift for nursing research grounded in a critical ethics of care and emancipatory knowing. By articulating the concept of embodied non‐belonging through a contemporary posthumanist geographical lens, this paper argues how migrant health emerges or deteriorates through complex relational dynamics between bodies, spaces, and affects. While recognizing that non‐belonging may sometimes be actively sought or strategically embraced, the analysis focuses on its production as a form of embodied suffering within hostile socio‐material assemblages. Specifically, our theoretical analysis demonstrates that migrants' experiences of non‐belonging and health are corporeally inscribed through three key more‐than‐human and more‐than‐representational processes: (1) unstable interactions in material assemblages, (2) open vital bodies under environmental influences, and (3) immediate spatiotemporalities arising from pre‐reflexive events. Within this framework, nursing acts as a mediator of spatial assemblages, participating in a constitutive “taking‐place” of health, from migration policies to micro‐interactions. This framework calls for rethinking nursing practice as an active posture of attending to and reconfiguring exclusion‐generating environments, thereby embracing health's profoundly relational and political dimension.

## 1 Introduction

Health inequities affecting migrant populations manifest themselves through various interconnected factors: restricted access to healthcare due to linguistic and administrative barriers, precarious housing and employment conditions, compromised mental health linked to trauma or anxiety, heightened vulnerability to infectious diseases, inadequate healthcare systems, challenges in managing chronic conditions, and systemic stigmatization through racism and xenophobia (World Health Organization 2022, 2024). These factors produce multidimensional precarity that extends beyond access to medical services, rooted instead in structural exclusion dynamics where legal, social, and cultural factors play a pivotal role. This exclusion often manifests itself in a deep sense of not belonging (Lähdesmäki et al. 2014; Mattes and Lang 2021). When exclusionary conditions persistently produce the feeling of needing to be elsewhere, it can lead to profound existential alienation: individuals gradually lose their spatial and sensory references, and their sense of connection to the world fractures. This can have a detrimental effect on physical and mental health (Lems 2016; Wise 2010).

The process echoes what Goffman (1961), in a different context, termed the mortification of the self, a systematic stripping away of previous social anchors. In the asylum‐seeking context, exclusionary practices can approximate the logic of total institutions (Quirk et al. 2006; Schliehe 2016), making this concept a useful lens for tracing the erosion of belonging. Because this erosion operates through mundane, relational, and often pre‐reflexive processes, it remains elusive to traditional analytical frameworks, which, being often overly categorical or static, fail to grasp their processual and relational dimensions, and how these dynamics together concretely shape bodies and lived experiences. The situation is further aggravated by technocratic health policies that prioritize quantification and isolated determinants (Andrews 2019; Cummins et al. 2007). Such policies exemplify the broader inability of categories to account for the configurations they are meant to describe. This inability resonates with findings from the International Organization for Migration (2021, 2024) and the World Health Organization (2022), as they identify the intricate entanglement of health determinants, vulnerability, and intersectionality as sources of structural disparities. While this factor‐based approach is undeniably valuable for identifying broad inequities, it still falls short, in our view, of capturing the granular complexity of lived experiences. This gap is epistemological, stemming from and maintained by a substantialist ontology that regards categories as pre‐existing and relatively stable entities (Guba and Lincoln 1994).

Yet, exclusion is not simply a matter of administrative categorization. It is also an embodied experience. Systemic exclusionary dynamics generate active processes that leave their mark on bodies and experiences, altering health states (Andrews 2019). Understanding exclusion as a corporeal reality that is lived, felt, and somatically registered therefore demands a theoretical lens attuned to process and rupture. This aligns with the broader shift in migration studies from identity to belonging, which better captures dynamic attachments (Lähdesmäki et al. 2014; Yuval‐Davis 2006). However, as Lähdesmäki et al. (2014) note, research on belonging often retains a positive bias, focusing on integration while marginalizing the experience of failure and exclusion. Furthermore, they call for a more rigorous theoretical engagement beyond descriptive empiricism. The notion of embodied non‐belonging (Mattes and Lang 2021) directly addresses these gaps. Serving as a multi‐dimensional analytical and heuristic perspective, it brings to the fore the entangled dimensions through which exclusion causes existential and health‐related ruptures. Following Csordas (1990), we understand embodiment as the dynamic process emerging from the synthesis between *habitus* (Bourdieu 2008), as embodied practice, and the *pre‐objective*, as *pre‐reflexive* perception, which positions the body as the existential ground of culture and self. This paradigm foregrounds the pre‐reflexive, corporeal and socially constituted conditions of experience (Cromby 2014), which are crucial for understanding how social exclusion is not only experienced but also internally registered and lived as a somatic reality. The phenomenological inheritance of the intentional, world‐oriented body‐subject, as described by Merleau‐Ponty (1976), cannot fully explain the processes through which such embodied states are produced. These processes cannot be adequately grasped if the entities involved, such as bodies, spaces and affects, are conceived as pre‐existing substances. Addressing them requires moving from the level of the concept to the level of ontology. We therefore argue for a shift to an ontology of difference (Deleuze 2013), in which the pre‐personal is no longer intentional but an impersonal field of affects and intensities, being is not substance or identity but difference, multiplicity and becoming, and identity is an effect rather than a starting point. This ontology conceives being as a perpetual becoming in which phenomena are not pre‐existing entities but multiplicities that emerge through dynamic relations and assemblages (Duff 2014). This shift redirects the analysis toward the processes that give rise to embodied states. Yet the spatial and affective dimensions through which these states take shape remain undertheorized. A framework attuned to these dimensions is required to capture the relational processes through which non‐belonging emerges. We contend that a contemporary geographical approach offers precisely this.

Contemporary geographical theories, which mobilize Deleuzian concepts within a situated social‐scientific register rather than reproducing Deleuze's philosophy as such, build upon a relational understanding of space (Merriman 2022; Murdoch 2005). This approach rejects static, singular conceptions of space, prioritizing the forces and relations through which space is constituted in plural and processual productions (Thrift 1996). It is also interested in the intimate textures of everyday life (Anderson 2016). While non‐representational and posthumanist approaches have flourished within contemporary human geography, the health sciences, as D. Holmes et al. (2024) point out, have been slow to embrace posthumanist ontologies, largely remaining tethered to a humanist conception of the individual. Andrews and Duff (2019a) have articulated a posthumanist variant of this geographical tradition specifically for the study of health, offering a framework that directly addresses this existing gap.

To analyze how non‐belonging is produced through relational processes and spatial configurations, we therefore bring the concept of embodied non‐belonging into dialogue with this approach. This dialogue traverses the traditional dichotomies such as human/non‐human, natural/artificial, body/mind, exposing the porosity of boundaries between bodies, affects, technologies, texts, and environments. Moreover, it understands health through more‐than‐human assemblages, making visible how legal, spatial, and affective elements interact to shape migrant health, exposing the often invisible yet powerful mechanisms of social and healthcare exclusion. Articulating embodied non‐belonging with this contemporary geographical approach thus allows us to conceptualize migrant health not as a sum of fixed biological, psychological, and social factors but as a relational phenomenon emerging from interactions between bodies, affects, and inhospitable or hostile environments.

Echoing Philo's (2017) call for “less‐than‐human” geographies that are attuned to what diminishes, curtails, or destroys human capacities rather than what enhances them, as well as the critique of the positive bias in belonging research (Lähdesmäki et al. 2014), our analysis focuses primarily on the destructive side of non‐belonging. This follows a critical tradition that examines the more‐than‐human functioning of institutional power and its often harmful consequences (Andrews and Duff 2019a; Dekeyser et al. 2022, 2024; Philo 2012, 2017; Rich 2010). To demonstrate how this perspective illuminates the socio‐material production of non‐belonging, we draw on a series of illustrative cases from recent migration studies. Building on the thematic volume on embodied belonging (Mattes and Lang 2021) and its ethnographic contributions (Bendixsen 2020; Brenman 2021; Von Poser and Willamowski 2020), we selectively expand to include research that documents the embodied contours of migrant exclusion across different European contexts (Grønseth 2018; Huizinga and Van Hoven 2018; Subramani 2024). These cases help to unfold the implications of our perspective by grounding it in concrete spatial and affective dynamics.

By articulating embodied non‐belonging with a contemporary geographical perspective, this article develops an analytical lens to uncover the socio‐material mechanisms through which healthcare exclusion is assembled, embodied, and felt, providing a critical foundation for rethinking nursing practice with migrant populations.

## 2 The Limits of Deterministic, Humanist, and Constructivist Approaches to Embodied Non‐Belonging

### 2.1 An Epistemological and Ontological Aporia

Traditional analytical frameworks, despite their contributions, produce effects that call for systematic critique. While these have contributed to exposing health inequities, their rigid application often reifies fluid realities into fixed categories. As Andrews (2018) notes in his reading of Duff (2014), this results in a proliferation of “numerous other concepts with their own definitional and implementation challenges” (p. 29) that circulate without producing transformative effects on the structures they describe. This rigidity is particularly evident in positivist‐inspired treatments of health determinants, whether economic, environmental, or social, which are often isolated within linear and decontextualized analytical grids. Such a static approach rests on the problematic idea that these determinants are objective and stable, when in reality, their nature and effects are fundamentally contingent (Andrews 2018; Duff 2014). Duff (2014) calls for replacing “the notion of context with that of the assemblage,” arguing that “nothing is *determined* in life” (in Deleuze 1978) and that there is “no sense distinguishing health problems from their social contexts” (p. 3). This shifts the focus of analysis from fixed determinants to the dynamic processes through which health emerges or deteriorates via complex interactions between individuals, institutions, and environments. The aim is to resituate the subject within a veritable matrix of social, political, and economic forces, where context is understood as a combination of dynamic spaces, bodies and affects, rather than as a sum of disconnected elements (Duff 2014).

This shift in perspective also demands a critique of humanist and social constructionist approaches to health and illness, which struggle to fully grasp the complexity of embodied health and obscure the pre‐personal and more‐than‐human dimensions that actively participate in its production. Humanism, by positing the rational individual as the ontological foundation of progress, deliberately ignores the material, affective, and relational forces that nevertheless transcend and constitute human experience. This critique has a long genealogy, from Heidegger's (1998) dismantling of humanism as metaphysics to Foucault's (2012) announcement of the “end of man” and Horkheimer et al.'s (2002) critique of instrumental reason. More recent work in non‐representational and posthumanist geographies (Anderson and Harrison 2016; Andrews 2019; Braidotti 2013) extends this critique to the study of health and space. Social constructionism, for its part, by reducing reality to mere discursive constructions, risks not only minimizing the agency inherent in matter and environments but also producing a devitalized analysis of the processes through which inequities emerge (Anderson and Harrison 2016; Andrews 2018; Duff 2014). Yet it is precisely this inscription in the body and space that grants non‐belonging its persistence and its particularly damaging effects on migrant health.

These significant limitations call for the development of a framework capable of fully capturing the processual and embodied dimensions of exclusion. Here, a contemporary geographical perspective, focused on the relations between bodies, spaces, and affects, provides the needed heuristic. Such an approach offers a means of accounting for the social in a “radically” constructionist manner, as opposed to a social constructionist perspective (Anderson and Harrison 2016). Andrews (2017) emphasizes that its value lies in demonstrating “how the physical and sensory overflow and interplay with conscious, personal, social, political, economic, and institutional processes, and how both levels contribute to power, significance and meaning in life” (p. 3). This geographical perspective proves particularly well‐suited for analyzing the “taking‐place” in nursing spaces, enabling the capture of lived experience without lapsing into excessive anti‐humanism or implicit normative humanism (Anderson and Harrison 2016). The perspective addresses three central questions: (1) How do meaning and significance emerge from practical action? (2) How is action organized within more‐than‐human configurations despite the contingency of social orders? (3) And how can events transform existing orders? This approach thus paves the way for a more nuanced understanding of the multiple dimensions that shape migrant health.

### 2.2 From Categorical Thinking to Critical Effects: The Politics of Conceptual Reification

The epistemological and ontological limitations critiqued above materialize in the political and moral effects of the conceptual vocabulary they enable. While deterministic, humanist, and social constructionist approaches differ in their areas of focus, their collective blind spots allow a series of categorizations or key concepts to be deployed in ways that perpetuate the conditions they seek to describe, obscuring the relational production of health and exclusion. Migration status offers a particularly salient example. Categories such as “refugee,” “asylum seeker,” or “undocumented migrant” act as socially, politically, and legally constructed classifications that organize differential access to care, housing, employment, mobility, and social protection. The difficulty lies not in categorization as such, but in the ways these classifications are enacted and thereby reified as fixed and self‐evident, structuring inequitable relations. As S. M. Holmes et al. (2021) and Willen (2012) have shown, these categories do more than describe legal positions. They carry moral weight, shaping which spaces may be entered, which rights become accessible, and whose suffering is legible as a claim to care.

One critical pitfall of this broader categorical logic is the conflation and reification of precarity and vulnerability. Following Butler (2016), vulnerability must be understood as a fundamental, ontological condition of relational, embodied existence, and our openness to being affected. Precarity, by contrast, designates the politically induced and unevenly distributed condition in which specific populations are systematically exposed to harm, suffering, and death due to failing social and economic networks. In dominant migration discourses, the static, categorical use of vulnerability often collapses this distinction by portraying people who migrate as an inherently at‐risk group. This not only obscures the specific assemblages of policies, legal barriers, and hostile environments that actively produce their precarity, but also, as Butler (2016) warns, risks reinforcing a paternalistic logic that positions migrants as passive subjects in need of protection, thereby effacing their agency and modes of resistance.

Similarly, the discourse of resilience, in its popular deployment, can function as a governmental technology (Joseph 2013) that individualizes the immense effort required to endure oppressive systems. This process is not unique to resilience. It mirrors the reterritorialization^1^ of other emancipatory care concepts, such as the recovery model in mental health, which has been stripped of its radical potential and repurposed as a normative, institutional tool (Johansson and Holmes 2023). In both cases, the focus shifts from the socio‐material assemblages that exhaust bodily capacities to an individualized narrative of adaptation, thereby naturalizing the structural violence that necessitates such endurance in the first place. Likewise, when trauma is viewed as a discrete psychological event within an individual, it risks pathologizing the survivor while obscuring the ongoing political violence and social injustice that constitute the traumatic reality. A relational perspective, in contrast, would understand trauma, not as an internal pathology, but as a somatic resonance of injustice, continually reconfigured through hostile encounters (Helbich and Jabr 2022).

A related concern applies to intersectionality, which remains an indispensable analytical tool for understanding how identities are shaped by intersecting systems of domination, such as race, gender, class, and migration status, and how these systems produce specific and cumulative effects that multiply vulnerability. Foundational intersectionality scholarship (Crenshaw 1989, 1991) was explicitly developed to challenge static and additive understandings of social categories and identity, insisting that the forms of discrimination thereby created are not reducible to the sum of their components. Our concern is therefore not with intersectionality as such, but with its mechanical application as a layering of fixed categories. Applied without due care, it misses how identities are dynamically activated and experienced in different material spaces. As Ahmed et al. (2003) show, migrant identity is constructed through dynamic interactions between social markers and material spaces. A body perceived as “foreign,” for instance, will be perceived differently depending on whether it is located in a reception center, a cosmopolitan neighborhood, or a hospital ward. This relational fluidity eludes purely categorical models, which, by treating identity as a static grid, can render invisible the very power structures that intersectionality aims to critique (Saldanha 2016).

This categorical logic finds its ultimate expression in the ideal of inclusion, which often presupposes a stable social body into which migrants must be incorporated. This static framing obscures its own constitutive paradox: the act of including some necessarily defines and excludes others, thereby reinforcing the boundaries it purports to transcend (Ahmed 2012). Even the practice‐oriented notion of “doing inclusion” can be stripped of its political force when treated as a predefined objective, masking how such efforts may inadvertently reproduce relations of dependency and unchallenged hierarchy (Kangas‐Müller et al. 2024). Inclusion is therefore not the alternative to exclusion, but its reconfiguration.

This categorical framing rests on a deeper ontological configuration. Drawing on Searle (1983, 1995), this configuration can be understood as resting on a Network of assigned statuses, a shared Background of tacit understandings that give meaning to categorical labels, and the Intentionality of individuals who navigate within this system. Searle's analysis is descriptive rather than critical: our aim here is to mobilize its vocabulary while reversing its normative orientation. Together, these elements sustain a worldview in which health and identity appear as properties of pre‐constituted individuals rather than as relational accomplishments. Operating as a regime of truth (Foucault 1980), this configuration legitimizes specific forms of knowledge (e.g., clinical, epidemiological, and administrative) and produces corresponding subjectivities (e.g., the vulnerable migrant, the resilient survivor), which individuals are then invited to inhabit. Furthermore, this logic functions as a territorialization (Deleuze and Guattari 1980) that captures the processual, complex realities of migrant trajectories and striates them into fixed, manageable categories for *government*, intervention, and knowledge production purposes (Dablemont 2026, forthcoming). This striating process creates a rigid grid of thought and practice that conforms to institutionalized rules and norms. It opposes the free flow of ideas and reinforces a regime of appropriate knowledge (D. Holmes et al. 2007).

It is within this naturalized worldview, or regime of truth, that a tacit moral reckoning of deservingness becomes operational. As critical research on migrant health articulates, this concept emphasizes “the ways in which assumptions about whose health deserves attention and care influence every aspect of healthcare provision” (S. M. Holmes et al. 2021, 2). Defining such concepts as stable attributes risks turning them into filters for moral assessment rather than tools for understanding. This logic operates as a material‐discursive practice that converts administrative and clinical categories into hierarchies of human value, shaping access and treatment (S. M. Holmes et al. 2021; Willen 2012). This reification constitutes a form of epistemological violence (Teo 2010). The primary manifestations of this violence are the production of fixed, often pathologized identities, and the naturalization of exclusion as a personal or cultural deficit rather than a politically orchestrated outcome (Butler 2016; Teo 2010). A rigid conceptualization of identity thereby enforces a false sameness, masking the dynamic, power‐laden processes of belonging and non‐belonging through which inclusion and exclusion are lived and felt (Brubaker and Cooper 2000; Lähdesmäki et al. 2014; Yuval‐Davis 2006).

Collectively, by neglecting more‐than‐human and more‐than‐representational dimensions, traditional frameworks prove incapable of grasping how non‐belonging is inscribed in material and affective processes that far transcend consciousness and language. Embodied non‐belonging names the process through which exclusion is inscribed in the body, shaping lived experience at the intersection of the epidemiological, the phenomenological, and the spatio‐sensorial (Mattes and Lang 2021). For migrant populations, this non‐belonging translates into specific effects and affects, such as hypervigilance during police checks (De Genova 2002), frustration and psychological pressure during asylum procedures (Willen 2021), or persistent discomfort in spaces ostensibly designed to be inclusive (Morrison et al. 2020). Though difficult to quantify or qualify, Mattes and Lang (2021) argue that these experiences generate a perpetual tension, which is particularly detrimental to physical and psychological well‐being. They suggest that a fundamental rethinking of research must focus on the complex ecology of relations between bodies, spaces, and public policies. As Willen (2012) argues, “classificatory terms are useful only to the extent that they help us gain insight into the specific social, political, and epidemiological *processes* that shape the health circumstances of individuals and groups” (p. 810).

The task, then, is not to abandon these conceptual tools, but rather to reconfigure them radically, so that they attend to processes rather than reify states. Adorno (1983), writing from within the tradition of critical theory, calls this gesture constellation,^2^ a way of holding concepts together without subsuming them under a unifying abstraction. His gesture remains dialectical and critical‐humanist; it is not an ontology of immanence. Yet it captures precisely what we seek. We therefore retain the gesture while departing from its dialectical framework.

The unifying moment survives without a negation of negation, but also without delivering itself to abstraction as a supreme principle. It survives because there is no step‐by‐step progression from the concepts to a more general cover concept. Instead, the concepts enter into a constellation. The constellation illuminates the specific side of the object, the side which to a classifying procedure is either a matter of indifference or a burden.

(p. 162)

As Andrews (2018) puts it in his reading of Duff (2014), academics might be better off “rather than thinking about what health is, thinking about how health arises” (p. 29). We now turn to this precise task, proposing a contemporary geographical framework designed to illuminate the processes through which migrant health and embodied non‐belonging emerge within dynamic socio‐material configurations.

## 3 Migrant Health and Posthumanism: The Threefold Emergence of Embodied Non‐Belonging

To address this need for a processual lens, we ground our analysis in the geographical theoretical framework developed by Andrews and Duff (2019a). Consistent with the anti‐reifying critique above, their approach redefines health as an emergent property where the person is not a stable entity, but a process of becoming, constituted through affective encounters and assemblages (Deleuze and Guattari 1980; D. Holmes et al. 2024). As an established explanatory framework focused on the processual emergence of health, it builds on a plane of immanence^3^ whereby health and well‐being emerge in spatial and affective relations amongst material entities. This is not a reproduction of Deleuze's thought as such, but a social‐scientific deployment of it: the framework retains the immanentist orientation while operating at the level of empirical analysis rather than metaphysical speculation. It is durable and assistive, providing clarity on the fundamental processes always at work in such emergence, albeit with infinite possible empirical expressions. The framework, or close variations, has been deployed in the context of whole onflow and the plane of becoming, a conceptual addition to the Deleuzian plane of immanence that captures life's open‐ended emergence (Andrews et al. 2026). It has also been deployed in critical gerontology and emergent aging (Andrews and Duff 2019b), in social work research and the emergence of practice (Andrews 2020), and in posthumanism and health geography (Andrews 2019; Andrews and Rishworth 2023).

Two concepts central to this framework require clarification. The first one is “affect,” which Anderson (2016) describes as an infectiousness whereby interacting bodies—human and non‐human—affect one another, either energizing one another and increasing their powers and capacities for acting (positive affection) or de‐energizing one another and reducing their powers and capacities for acting (negative affection). Affect is registered by human bodies as a vague but powerful sensation; positive affect feeling like a “passion” and affinity during involvements, negative affect feeling like a toxin and disassociation during involvements. Either way, affect is felt often as the body's part in, and relation to, prevailing atmospheres and bigger pictures. In terms of well‐being, affect can involve an immediate sense of “joy” or “sadness”—as Deleuze, reading Spinoza, would have called it—when experienced in and of itself. Secondarily though, as a force, affect might impel bodies into or out of involvements in certain types of activities that themselves might be health and well‐being generating or detracting. Critically, particular affects might be generated by political or commercial interests, enrolling bodies, and excluding some, across numerous public and private spaces and areas of life. The second concept is “assemblage.” Defined by Deleuze and Guattari (1980), “an assemblage is precisely this expansion of dimensions within a multiplicity that necessarily changes in nature as it increases its connections” (p. 15; author's translation). As they insist, there is “no machinic assemblage that is not a social assemblage, no social assemblage that is not a collective assemblage of enunciation” (Deleuze and Guattari 1975, 147; author's translation). Assemblages thus operate through desire as a circulating flow that produces connections, applying to structures from individual behavioral patterns to institutional organizations and spatial arrangements. In this sense, an assemblage is never merely present but draws on accumulated pasts while anticipating future becomings (Rose 2016).

To grasp this processual and affective constitution of health, Andrews and Duff's (2019a) framework captures three interdependent processes that reveal how non‐belonging is not merely experienced but embodied through more‐than‐human interactions: (1) health as emergence of relational material assemblages that actively reconfigure bodies, (2) health as emergence of open vital bodies that somatically register these transformations, and (3) health as emergence in immediate, pre‐personal spatiotemporalities where mutations become embodied at infra‐conscious level. Together, they dismantle the notion of health as a static condition, reframing it instead as a continuous process of corporeal and affective mutations negotiated within specific socio‐material configurations. We unfold these three processes below, drawing on empirical studies that explore the embodied contours of non‐belonging among migrant populations.

### 3.1 Health as Emergence of Relational Material Assemblages

The first process radically redefines health: it is no longer an intrinsic property of human bodies, but the dynamic expression of specific assemblages in which humans and non‐human entities (e.g., objects, institutions, discourses, affects, and technologies) are inseparably entwined. These unstable configurations co‐produce states of health or illness through contingent interactions, where each element, human or not, manifests distinct relational agency. This raises the question of which elements interact and how in a given context.

For migrant populations, these assemblages incorporate diverse elements: the specific materiality of reception centers, the administrative documents that testify to their legal existence, the public transport network they navigate, a translation app on their smartphone, care *dispositifs*, and affects like fear or insecurity—each marked by prior relational histories. Precarious states of health emerge not through isolated causality but through their dynamic configuration (Andrews 2019; Duff 2014; Whatmore 2006). Consider Ferhat, a Syrian refugee in the Netherlands, who wishes to study economics (Huizinga and Van Hoven 2018). He stands before the university library:

Many students are in the library for studying or reading. I want to read in the library, but I can't go in the building, because I'm not a student yet.

(p. 314)

Ferhat does not have a student card, yet the turnstile and the card reader stand before him regardless. In this assemblage, the turnstile condenses a policy that leaves him in limbo, producing a barrier that is both material and existential.

While the turnstile is material and unambiguous, exclusion can also take more subtle, interactional forms. Seema, a Kurdish woman requiring orthopedic care in Zurich (Subramani 2024), has a specific and recurring need: a childhood fire accident resulted in the amputation of two toes, necessitating regular consultations for specialized footwear. What she carries is the memory of being rendered invisible by Swiss institutions: during her brother's hospital stay, for instance, she was treated as if she did not exist. Having arrived in Switzerland as a child, she has seen such moments sediment, over the years, into a durable sense of being othered, an alienation that now accompanies her in these healthcare encounters. It is this embodied history that enters the consultation room:

The third time I've tried to get the right footwear; he doesn't listen to my needs. I had to explain myself so many times… but he just eye rolls. He doesn't even touch my feet or help me with the right fitting.

(p. 1951)

The clinician's eye roll and refused touch are not isolated interpersonal failures. They are based on ideas about who should receive care, whose body is suitable for touch, and whose pain is important. These ideas are influenced by migration discourses, institutional practices, and histories of racialization and gender. The consultation room is an assemblage where these histories converge, and where Seema's body is made to feel its place.

If assemblages can be silent machines of exclusion, they can also be sites of deliberate, reflexive intervention. Consider the funeral assembled by a Tamil community in northern Norway following the accidental death of Bala, a young worker at the fish plant (Grønseth 2018). His friends gather to plan the ceremony. They are afraid not only of Bala's ghost, which they hear knocking on doors and barking like a dog, but of something else. Thore, a friend who has known Bala for over a decade, says:

We are afraid of doing our things […] We do not want to be exotic [in a way that] they can ridicule and harass [us]. We want to have a funeral from which Bala and his family can find peace. But, we do not want to have a funeral that creates a greater gap between Tamils and Norwegians. The gap will be filled with racism. We do not want that.

(p. 2622)

The assemblage they construct includes a Protestant church, a Tamil Catholic priest who knows Hindu songs, a procession through the village, the open coffin, and the young widow, Janisha, whose fate is bound up with the *tali*, the gold necklace she must decide whether to return to the coffin, in a ritual that condenses the weight of tradition, community belonging and gendered expectation. The assemblage also includes Norwegian coffee, cake and a memorial speech. Every element is weighed against its potential to either narrow or widen the racialized gap. Here, the capacity for collective mourning and post‐funeral peace depends entirely on the assemblage's configuration.

Read through the lens of this article, Grønseth's (2018) rich ethnographic account reveals a deeper structure that remains implicit: the political economy that drives migrant bodies into dangerous sectors where risks are borne by those recruited precisely because they will accept what others refuse. From this perspective, non‑belonging is not only an affective experience but also a structural condition that results in bodies being deemed less worthy of protection. Although the funeral was not intended as a political act, it nevertheless contends with this structure, the “gap” that Thore feared would be “filled with racism,” whether its architects acknowledge it or not.

From the turnstile that excludes to the funeral that negotiates belonging, these examples have illustrated the diversity of elements that compose such assemblages and the work of assembling them. It is to how they actually act on and through bodies, enabling some capacities, constraining others, that we now turn.

### 3.2 Health as Emergence of Open, Vital Bodies

The second process redefines health as emerging in open vital bodies, conceived not as stable entities nor as the bearers of fixed identities, but as shifting convergence points (Andrews and Duff 2019a; Grosz 2017), perpetually reconfigured through material and affective encounters (Andrews 2018; Andrews and Duff 2019a). This conception of the body as “living archive” of past and present affects (Andrews and Duff 2019a) entails continuous absorption and reaction to environmental forces, generating intrinsically unstable health states.

In the environments migrants navigate, these forces are often hostile. Bendixsen's (2020) ethnographic study of irregular migrants in Norway documents how the denial of asylum becomes internalized. Her participants describe the effects of prolonged exposure to legal exclusion, the somatic registration of a politically induced condition of non‐belonging, as “[h]eadaches, stomach aches, pain, lack of appetite, mental torture, depression, becoming mad, and losing one's mind […]” (p. 487). As Zoya, an Ethiopian woman, explains:

[…] [W]e are sandwiched. In our country there are problems […] Here, we are denied […] So, we are frustrated, we get mental torture, we are physically damaged. Some of our friends, some of our people, they got crazy, they become mad, some of them commit suicide […] You are depressed and depressed and depressed.

(p. 487)

Zoya also articulates the cruel paradox at the heart of this process:

After you go mad, they want to protect you. That's stupid. After I lose my mind, if they give me gold or if they gave me anything, it's meaningless for me. I need it when I'm healthy, when I can work, when I can do something. But after I am useless and mad, they will give me protection, it is meaningless.

(p. 487)

The system is structured so that recognition, and with it, protection, is only activated once bodies are already broken, exposing a logic that turns categories of vulnerability into instruments of exclusion rather than care. Zoya's testimony makes clear that letters of rejection, the loss of employment and healthcare rights, or the inability to afford treatment are not merely external circumstances. They penetrate the body, progressively reducing its ability to act, hope, and live. But this contraction of vitality is not always visible. As Bilaal, a Palestinian in his twenties, observes:

It's like in jail, […] when you are in jail and they want to torture you […] they don't make marks on your body. […] And Norway is doing the same thing. They punch you in the face, but in a way that is not obvious.

(pp. 489–490)

The body becomes an archive of wounds that leave no external trace. Yet, these invisible inscriptions profoundly diminish the body's capacity to engage with the world to trust and to project itself into a future. The violence of non‑belonging operates not only through what is done to the body but through what is withheld, such as recognition or care.

If Bendixsen (2020) reveals the slow, cumulative inscription of structural violence, Subramani's (2024) study of migrant women in Zurich captures how such inscriptions are activated and intensified in the immediacy of a clinical encounter. Mala, a Tamil woman from India, arrives at a medical appointment after waiting for weeks. For this consultation about her chronic headaches, she has prepared with extraordinary care:

I dressed jummennu (clothed well) with perfume […] I didn't dress up like I was meeting a doctor […] I took headshower […].

(p. 1952)

Mala comes from an upper‐caste background but experiences discrimination based on skin color in a white‐dominated environment. Her meticulous preparation is not vanity. It is a labor of self‐presentation aimed at reconfiguring the terms of the impending encounter, an assertion of dignity that pre‐empts the gaze that might reduce her to a “mere foreigner.” Yet the encounter with the practitioner unfolds otherwise:

It is all about colour for them […] she gave her hand just like, like this (demonstrated with touching few finger tips) as if I am *sakadai* (sewage)… she didn't even touch me… stood in distance… didn't come near me…

(p. 1952)

The clinician's gesture is perceived as the annihilation of Mala's labor of dignity. Despite her best clothes, despite her careful preparation, her body is rendered unworthy of human contact. The clinical encounter does more than fail to care: it actively wounds, leaving a trace in the diminished capacity of her body to seek future care. This experience of being wounded is not hers alone. Subramani (2024) shows that what is archived here is not only individual wounding but a shared knowledge of what it feels like to be constantly treated as inferior, transmitted through the very experiences that contract vitality. As another participant, Mani, reflects:

[W]ell when we take shit we know how it feels… so you should make sure we respect each other… but a bit of love, a bit of care that is also needed for all strangers like us too.

(p. 1954)

This shared knowledge materializes in concrete acts of care among migrant women in Zurich. Such practices take shape through consistent gestures. As Kannagi explains:

We all are dealing with these “auslander” experiences here… sometimes we laugh, sometimes we cry, but we are there for each other.

(Subramani 2024, 1954)

This “being there” for each other is not a vague sentiment but a practical, embodied responsiveness that momentarily restores a sense of vitality and connection. By showing up at a friend's workplace to cover a shift, or spending visiting hours at a hospital simply to be present, these women enact a relational practice where recognition of shared vulnerability becomes the ground for mutual care.

In a different register, Huizinga and Van Hoven's (2018) study of Syrian refugees in the Netherlands shows how men perform a similar reconfiguration through the shared meal. Ferhat, the same student who could not enter the university library, travels half an hour by bus with friends to a butcher that meets their dietary requirements, dividing ten kilos of meat among themselves. He explains:

Actually I quite miss this habit from Syria. I am used to eating with my whole family. We were always eating together… every day. But then I was living alone and I was eating alone. Eating alone is not really a problem for me, but I also want to eat with other people. There are now two guys from Syria that I know here […] We cook for each other now […] You know, my heart is in Syria. By eating together, I kind of get a feeling of home.

(p. 315)

The shared meal reconfigures vitality through relation. It does not erase the distance between a heart still in Syria and a body learning to live in the Netherlands, but it momentarily bridges that distance, creating a pocket of belonging in an otherwise alienating environment. Temporarily released from the isolation of eating alone, Ferhat's body registers a feeling of home as a somatic affirmation that repair is possible, however fleetingly.

From Zoya's testimony of slow bodily erosion to Mala's annihilated dignity and Ferhat's brief respite during the shared meal, these examples have illustrated how bodies, viewed as living archives, can be sites where structural violence, micropolitics of care, and everyday practices converge. Physiology and emotions appear here as relational accomplishments (Andrews and Duff 2019a; Frost 2016), negotiated within assemblages that alternately wound and repair. The health states that result are always forms of vitality‐in‐tension. These encounters lodge in the flesh as “hauntings” that reactivate in contexts resonant with past injuries (Andrews and Duff 2019a; Andrews and Wilson 2020; Bissell 2016; Freedman 2017; McHugh 2009). Yet bodies appear neither as passive victims nor as heroic survivors, but as sites of continuous negotiation. It is to the immediate, pre‐reflexive spatiotemporalities where these mutations become embodied that we now turn.

### 3.3 Health as Emergence in Immediate, Pre‐Personal, and More‐Than‐Representational Spatiotemporalities

The third process focuses on the immediate spatiotemporal dimension of health, which often escapes conscious representation. Emphasized is the importance of the “taking‐place”—the event in its pre‐reflexive emergence, before linguistic or cultural codification. As explained by Andrews (2018), this approach centers on existence's “continuous flux,” capturing micro‐events, practices, rhythms, and atmospheres that traverse bodies before conscious awareness: “the physical, less‐than‐fully consciously felt, ever‐progressing leading edge of existence” (Andrews and Duff 2019a, 126). For migrant populations, this dimension is essential for understanding the raw, daily lived reality of non‐belonging.

Consider the London mental health clinic in Brenman's (2021) study. Mariam, an Eritrean woman, who fled trafficking and violence, now lives in dilapidated housing with a leaking roof. Her body, marked by these ordeals, carries this history as she crosses the clinic threshold. There, the waiting room itself becomes threatening: yellow walls meant to be cheerful convey emergency, an oversized buzzer controlling a door evokes imprisonment, sparse furniture amplifies discomfort. Mapping this space, Mariam does not simply describe it but performs it, taking on the role of the suspicious other patients: “Why are you looking at me? Are you going to beat me?!” (p. 29). Before any words are spoken, the body has already registered this space as unsafe.

Yet this same space produces a different non‐belonging for Dayo, a Nigerian woman who worked in investment banking before severe depression forced her to stop. For Dayo, the waiting room's tissues and water jug seem to demand: “Get crying!” (p. 31), an injunction to perform the vulnerable migrant she does not feel herself to be. The multilingual posters, intended to welcome, only reinforce her sense that this space is for others. The same material assemblage thus produces two different experiences of non‐belonging: imprisonment for Mariam, imposture for Dayo, each registering infra‐consciously before any clinical interaction. These experiences are intensified by the clinic's own precarity: forced to relocate due to rising rents, its limited budget constrains the possibility of creating a genuinely welcoming space, mirroring the precarity of those it serves.

This atmospheric dimension extends beyond institutional spaces. In the Northern Netherlands (Huizinga and Van Hoven 2018), Abdul‐Halim walks through streets empty of people:

We are not used to these empty streets. Talking to everyone on the street is typical Middle‐Eastern culture, but in the Netherlands I always have to look for social contact.

(p. 312)

The silence tightens around him. This is not merely an absence of encounter but an absence that accumulates, a slow contraction of possibility registered in the body. Yet if non‐belonging inscribes itself infra‐consciously, so too can its opposite. In the Turkish supermarket, Aziz is greeted by name, offered tea, invited to sit:

The supermarket is always very busy. Many people from different places […] They are talking, sometimes about war, sometimes about family. If I say hello, they say hello back […].

(p. 315)

Here, the body loosens into the familiar cadence of greeting and response. The supermarket functions as an assemblage calibrated to migrant sensory landscapes: the smell of familiar spices, the sound of Arabic, the ritual of tea‐drinking and conversation create an atmosphere of ease that bypasses conscious reflection.

But when the sensory landscape is unfamiliar, the same infra‐conscious register can produce acute discomfort. Von Poser and Willamowski (2020) describe elderly Vietnamese‐born therapy group members in Berlin, visiting a traditional German café, disoriented by unfamiliar table manners. One participant continued to ask about local table manners weeks later, revealing how the café's materiality had inscribed in his bodily archive as a lasting unease. Where the Turkish supermarket offers a sensory home, the German café demands an adjustment to codes that do not come naturally. The difference lies not in the spaces themselves but in how each assembles with the bodies that enter them, in whether the encounter expands or contracts the body's capacity to simply be.

This dimension also appears in therapeutic sensorial attunement. The same group of Vietnamese migrants in Berlin, survivors of war and displacement, and decades of social isolation, share a profound numbness, having lost the capacity to put suffering into words. For them, a visit to a tropical greenhouse becomes healing, not through explicit memories, but sensorially: the warm, humid air, the familiar scents, and the towering plants bypass conscious mind. The body, so often held in the tension, loosens.

But healing is not the only outcome of sensory immersion. During the same excursions, Mr. S photographed both a beautiful day and a spot without trees, the latter triggering war memories of demolished landscapes and corpses. His body tensed, his voice lowered, showing how the same environment could oscillate between reparation and wounding. The group's response smiles for the first photo, averted gazes for the second, revealing these spatiotemporalities as shared, held collectively. What Von Poser and Willamowski (2020) call “affective communities,” that is, moments when bodies synchronize sensorially without discursive mediation, emerge in such shared immersions, creating temporary pockets of belonging within otherwise alienating landscapes.

These examples reveal that the immediate spatiotemporality is never merely individual. The same waiting room can be experienced as a prison for Mariam and a stage for imposture for Dayo. The same sensory environment can offer repair or reactivate trauma, as Mr. S's camera shows. A street can contract a body through absence, and a supermarket can expand it through familiarity. What these diverse encounters share is that they are registered infra‑consciously. However, they are not purely personal but are shaped by forces that exceed the individual, such as housing policies, migration regimes, and funding cuts, which become palpable in the very atmosphere of a room, the silence of a street, or the discomfort of unfamiliar codes. It is this raw, daily lived reality, at once intimate and structural, that this third lens captures.

Together, these three interdependent processes reconfigure the understanding of migrant health, no longer as a state but as a continuous process of emergence and transformation, negotiated within material assemblages, registered in open vital bodies, and felt in the immediacy of spatiotemporal encounter. They reveal that non‐belonging is not just experienced but produced through turnstiles and averted touches, through empty streets and buzzing doors. Yet they also reveal that belonging, too, can be momentarily reconfigured, through the scent of a tropical greenhouse and the taste of a shared meal. These are processes that any critical nursing practice and ethics of care adequate to migrant health must attend to.

## 4 Discussion

The critical analysis developed in this article has identified a persistent gap in how migrant health and embodied non‐belonging are typically approached. Deterministic, humanist, and social constructionist frameworks reduce the environment to backdrop, categories to stable entities, and bodies to passive surfaces. What they miss is the active, constitutive role of space, matter, and affect in shaping health. A contemporary geographical perspective directly addresses these blind spots. Andrews (2016) traces the evolution of the nursing metaparadigm concept of environment from classical nursing theory to later spatial and social‐constructivist approaches, yet even these remain tethered to a humanist ontology that positions the environment as a mere backdrop. What remains undertheorized in nursing is the environment as active participant: an assemblage of forces, materials, and affects that produce bodies rather than merely surround them. As Willen (2021) observes, following Desjarlais (1997), to analyze embodied belonging and non‐belonging is to place ourselves “at the intersection of the phenomenal and the political” (p. 67). This is precisely what the three processes achieve: they reveal how exclusion takes place as phenomenal experience, felt in bodies, registered in the immediacy of encounter, and inscribed in living archives of affect.

The turnstile that refuses Ferhat entry, the waiting room whose yellow walls Mariam experiences as threatening, and the tropical greenhouse where Vietnamese elders momentarily release tension do not merely form the backdrop: they are assemblages that contract or expand a body's capacity to belong and to be well. These insights have direct implications for nursing practice, for the environment is no longer something nurses act within, but something they participate in reconfiguring. Their practice is guided by the questions that Lindell and Chinn (2024) place at the heart of emancipatory knowing: “What is wrong with this picture? What are the barriers to freedom? What changes are needed?” Emancipatory knowing refuses the false dichotomy between technical competence and the relational and political dimensions of practice, insisting that truly skilled nursing practice must identify and address the power dynamics that produce health inequities (Kagan et al. 2010).

If non‐belonging is produced in assemblages, then the nursing role requires shifting clinical attention from the individual patient to the assemblages that produce distress, from symptoms to the environments that contract or expand a body's capacity to act and belong. In this, the perspective may resonate with “neurodiverse” spatialities (Merriman 2022) and with Judge's (2018) neurodivergent perspective on sensing and space, where diverse ways of inhabiting space are understood not as deficits but as realities to be reckoned with. Embodied non‐belonging, in this light, may be understood as a lived reality that dominant regimes fail to make room for. As Althusser and Goshgarian (2015) remind us, scientific practices are never neutral: embedded in social relations, they risk reproducing the very order they claim to reform. Recognizing that the clinical is already political thus requires a critical impulse that prevents it from drifting into a purely affirmative register, leaving intact the existing order and its exclusions and hierarchies. Our deliberate focus on embodied non‐belonging rather than on more celebratory accounts of migrant agency or resilience is justified precisely by this attention to the negative (Dekeyser et al. 2022, 2024; Philo 2017). Emerging debates on “feeling out of it” (Andrews 2025; Bissell 2025) deepen this orientation from within health geography by calling for a non‐pathologizing focus on states of displacement, disengagement and dispossession, moving beyond the clinical, the individual, and the pathological to understand the spatial, affective, and ultimately political production of distress.

To care, then, is to cultivate what Antonsich (2010) might call a dual vision that sees in the intimate texture of a migrant's daily life (place‐belongingness) the political structures that shape it (politics of belonging). Farmer (2003) names the embodiment of the latter “pathologies of power” to describe large‐scale social forces that become sickness and suffering. This observation underscores the importance of framing distress as a relational outcome of historically marked bodies interacting with constraining environments, attending to how identities are dynamically activated in specific material places and to the somatic inscription of injustice (Ahmed et al. 2003; Helbich and Jabr 2022). Philo (2017) gives spatial precision to this understanding by distinguishing two interrelated dimensions of structural violence: “geopolitical violence,” the gradual accumulation of inequalities that produces premature deaths, and “violent geopolitics,” the manifestation of structural violence that “takes away, chips away, physically and psychologically, to leave the rags‐and‐bones (and quite likely broken hearts, minds, souls, spirits) of ‘bare life’ (after Agamben 1998)” (p. 258).

The effect of such dynamics is what Grosfoguel (2016), drawing on Fanon (1970) and De Sousa Santos (2007, 2010), theorizes as a global hierarchy between a “zone of being” and a “zone of non‐being.” This division determines whose suffering is acknowledged and whose is consigned to neglect. For bodies consigned to the zone of non‐being, the ethics of impotentiality (Dekeyser et al. 2024; see also Agamben 1999) is of particular urgency: it rejects the rehabilitative impulse to transform negative affects into positive ones and asserts the right to inaction when bodies are overwhelmed by structurally produced conditions. Read through Judge's (2018) neurodivergent lens, such inaction need not be interpreted as passive withdrawal. Instead, it can be understood as a more‐than‐verbal language of distress, a somatic response to hostile environments that operates according to a sensory system of communication that does not conform to the rules of verbal interpretation. This is a direct challenge to the ableist logic that measures human worth by one's capacity to act, to recover and belong and that systematically renders certain lives “less‐than‐human” (Judge 2018; Philo 2017). Recognizing this shared logic opens space for solidarity across distinct yet structurally connected forms of marginalization.

The cases examined in this article reveal this structural violence made flesh: the exhaustion of bodies, the annihilation of dignity and the imprisonment of the senses. This highlights the ethical demands placed on care. Caring, in this light, means refusing the technocratic depoliticization that reduces migrant health to measurable problems, the deservingness calculations that convert administrative categories into hierarchies of human value (S. M. Holmes et al. 2021), and the proliferation of empty concepts (Andrews 2018), while leaving untouched the assemblages that produce the need for care in the first place. It means understanding, with Zoya, that protection which arrives only after the body is broken is not protection at all, and with Mani, that “a bit of love, a bit of care” for “all strangers like us” is not sentiment but necessity.

## 5 Conclusion

The contemporary geographical perspective developed in this article proposes a radical reframing of the environment in migrant health, moving beyond its conception as a neutral backdrop or a simple collection of services. Integrating the concept of embodied non‐belonging (Mattes and Lang 2021) within an ontology of difference reveals health and exclusion as complex, dynamic constructions where juridical borders, clinical norms, material arrangements, and affective flows are inseparably entwined in the co‐production of lived reality.

By employing Andrews and Duff's (2019a) geographical perspective, articulated through three interdependent processes, we have demonstrated how non‐belonging (1) materializes in relational material assemblages, (2) is registered in open vital bodies, and (3) operates through immediate spatiotemporal experiences. This approach explains how marginalization becomes inscribed in and through bodies and spaces, transforming abstract social forces and categorical labels into a tangible, daily lived reality. Focusing on the “taking‐place” through micro‐events, interactions, and affective charges allows us to explain the social production of health inequities rather than merely describe them.

This theoretical perspective opens concrete avenues for nursing research: documenting the material‐discursive assemblages that shape non‐belonging in clinical settings, examining migrants' bodily practices in relation to their environments, and capturing the pre‐reflexive affects that permeate care encounters. These avenues are not separate from practice. They converge on a way of attending to the situations nurses already inhabit, by investigating what assemblages are at work in a given space, what affects circulate there, and what is registered in the body before it becomes conscious or speakable, while remaining attentive to what categories are applied and what realities they leave out. Such questions disclose a nursing role mediating the spatial assemblages through which migrant health is constituted, from macro‐level migration policies to the micro‐geographies of the waiting room. The primary task is no longer to treat individual symptoms or vulnerabilities, but to intervene in the spatial assemblages that produce them. This requires a phronetic reorientation of attention, judgment, and action that moves beyond a technocratic vision which reduces migrant health to manageable variables, embracing instead its intricately relational and political character.

Ultimately, this perspective implies a paradigm shift in how nursing knows and acts, a shift that demands the deconstruction and reconfiguration of the institutional, spatial, and affective arrangements that generate exclusion. By revealing how embodied non‐belonging is continuously produced through invisible, socio‐material borders, this resolutely political approach provides a generative posture for navigating situated clinical realities, making room for diverse spatialities and paving the way for fairer, more responsive, and more equitable care practices.

## Disclosure of AI Use

The author, Julien Richard, declares the use of generative AI tools (DeepL Write v2, DeepSeek R1‐V4, Gemini Notebook 3.5) during the preparation of this manuscript in 2025–2026. These tools were utilized as cognitive and linguistic aids for the following specific tasks:

1. *Brainstorming and conceptual dialogue:* to identify the absence of tensions between the mobilized philosophical traditions, to test the congruence of the reflection with the theoretical framework, and to explore directions that resist established intellectual habits.
2. *Synthesis and literature processing:* to summarize and cross‐reference complex bodies of literature, facilitating the organization and synthesis of research notes.
3. *Translation and language editing:* to translate the author's French‐language first drafts into English and to refine the linguistic quality and readability of the text, as English is not the author's first language.

These tools were not used to generate original research data, to conduct the core analysis, or to write the manuscript autonomously. The conceptualization, argumentation, and theoretical choices emerged from the authors' collaborative dialogue. The first author (J.R.) critically reviewed and verified all AI suggestions, retaining full intellectual control over the final content. The co‐author (G.J.A.) did not use AI tools in the preparation of this manuscript. The manuscript does not involve human‐subject data.

## Funding

The authors have nothing to report.

## Ethics Statement

The authors have nothing to report.

## Conflicts of Interest

The authors declare no conflicts of interest.

## Data Availability

The authors have nothing to report.

## Footnotes

1 For Deleuze and Guattari (1987) in Parr (2010), deterritorialization refers to a movement producing change that frees up fixed relations, exposing a body to new organizations. Reterritorialization is the process by which these deterritorialized flows are recaptured and fixed within normative frameworks.

2 Adornian constellations prevent the reification of concepts by holding them within a network of relations that is constantly reconfigured (see Adorno 1983, 162–166).

3 Deleuze and Guattari (1994) describe the plane of immanence as “a radical empiricism” that “presents only events, that is, possible worlds as concepts, and other people as expressions of possible worlds or conceptual personae” (p. 49).
